# Epilepsy and Other Neurological Complications in Pediatric Patients With Hemophilia Complicated With Intracranial Hemorrhage: A Retrospective Case Series

**DOI:** 10.7759/cureus.74261

**Published:** 2024-11-22

**Authors:** Yuta Eguchi, Nobutsune Ishikawa, Yoshiyuki Kobayashi, Yoko Mizoguchi, Satoshi Okada

**Affiliations:** 1 Department of Pediatrics, Hiroshima University Hospital, Hiroshima, JPN; 2 Department of Pediatrics, Hiroshima Prefectural Hospital, Hiroshima, JPN

**Keywords:** epilepsy, hemophilia, intracranial hemorrhage (ich), neurologic complications, subdural hematoma (sdh)

## Abstract

Intracranial hemorrhage (ICH) is one of the most serious complications related to the prognosis of patients with hemophilia. It is also one of the major causes of epilepsy in general. However, there are few studies on epilepsy as a complication in patients with hemophilia. This observational study aimed to reveal the characteristics of hemophilia patients with epilepsy caused by ICH.

We retrospectively identified five patients with ICH (9.8%) out of 51 patients with hemophilia based on medical records at the Department of Pediatrics, Hiroshima University Hospital. Four patients (7.8%) had a clinical history of ICH, and one patient (1.9%) had no clinical episodes suggestive of ICH, with imaging findings indicating old hemorrhage. Two patients (3.9%) were observed to have focal epilepsy with tonic-clonic seizures, and both had experienced ICH. Both patients were well-controlled with oral sodium valproate. None of the five patients with ICH had other severe neurological complications, such as paralysis.

Epilepsy is a complication that should be recognized in patients with hemophilia, even in the absence of neurological sequelae such as paralysis. Imaging studies are important for patients with hemophilia and epilepsy, even in those without obvious clinical episodes of ICH.

## Introduction

Hemophilia A (HA) and B (HB) are X-linked inherited bleeding disorders caused by a deficiency of coagulation factors VIII and IX, respectively, with an estimated prevalence of 79-133 patients per million of the male population [1,2]. Although the advances in treatment, such as factor replacement therapy, have led to the improved life expectancy of patients [3], intracranial hemorrhage (ICH) occurs in 1.1-11.2 % of cases [4,5] and accounts for 14.1-18.8 % of the cause of deaths in patients with hemophilia even in recent reports [3,6]. In addition, ICH is a well-known risk factor for epilepsy [7-9], and there have been few reports of epilepsy complicated with hemophilia. The purpose of this case series study was to determine the rate of epilepsy, ICH, and other neurological complications in pediatric patients with hemophilia and to describe the detailed characteristics and outcomes of these epilepsies.

This study was approved by the Ethics Committee for Epidemiology of Hiroshima University (E2021-2727). This article was presented as a poster at the 64th Annual Meeting of the Japanese Society of Child Neurology on June 4, 2022.

## Case presentation

A retrospective case series based on medical records was conducted on patients with hemophilia whose last visit to the Department of Pediatrics at Hiroshima University Hospital was confirmed between 2016 and 2021. We examined histories of ICH, epilepsy, and other neurological complications. We identified five patients with ICH (9.8%), including four subdural hematomas (SDH) and one parenchymal hemorrhage, among 51 male patients with hemophilia (median age of 1.1) (Tables 1, 2). Four patients had a clinical history of ICH, and one patient was presumed to have ICH based on magnetic resonance imaging (MRI) findings after a detailed examination for afebrile seizures. Among the four patients with a clinical history, ICH occurred before they were diagnosed with hemophilia. All patients were diagnosed at less than two years of age, and four patients had severe hemophilia. They received appropriate coagulation factor replacement or emicizumab after diagnosis, and none of them experienced recurrence. Epilepsy was observed in two patients (3.9%) with ICH, whereas no epilepsy cases were observed in patients without ICH. Five patients (9.8%) had other neurological complications, all of which were neurodevelopmental disorders (NDDs) (Table 1).

**Table 1:** Characteristics of the patients ^†^The severity of hemophilia was defined by the plasma coagulation factor level as severe <1%, moderate 1-5%, and mild >5%.

Characteristics (n=51)	Values
Median age at the first visit, years (range)	1.1 (0-19.3)
Median follow-up period, years (range)	9.0 (0-21.2)
Hemophilia A, n (%)	36 (70.6)
Hemophilia B, n (%)	15 (29.4)
Severe hemophilia^†^, n (%)	36 (70.6)
Moderate hemophilia^†^, n (%)	5 (9.8)
Mild hemophilia^†^, n (%)	10 (19.6)
Intracranial hemorrhage, n (%)	5 (9.8)
Epilepsy, n (%)	2 (3.9)
Other neurological complications, n (%)	5 (9.8)

None of the five patients with ICH had severe neurological sequelae such as paralysis (Table 2). 

**Table 2:** Patients with intracranial hemorrhage ADHD: attention-deficit hyperactivity disorder; EEG: electroencephalography; VPA: sodium valproate; ^‡^treatment period in childhood met the inclusion criteria; ^§^Case 2 was considered to have a hemorrhage based on the old lesion observed on MRI.

Case	1	2	3	4	5
Age at inclusion (years)	13	20^‡^	1	9	13
Type of hemophilia	B	B	A	A	A
Severity of hemophilia	Severe	Severe	Severe	Moderate	Severe
Site of hemorrhage	Subdural left frontal	Right parietal^§^	Subdural left parietal	Subdural left temporal	Subdural right frontal
Age at hemorrhage (months)	9	-	0	4	13
Age at epilepsy onset (years)	3	10	-	-	-
Seizure type of epilepsy	Tonic-clonic	Tonic-clonic	-	-	-
Treatment for epilepsy	VPA	VPA	-	-	-
Prognosis of epilepsy	EEG normalized in 8 years	EEG normalized in 4 years	-	-	-
Other complications	ADHD	-	-	-	-

We performed statistical analysis using the chi-square test in Excel (Microsoft) to compare the type and severity of hemophilia and the rate of epilepsy and NDDs between patients with or without ICH. Statistical significance was set at p<0.05. There were no statistically significant differences in the type or severity of hemophilia. However, the rate of epilepsy was significantly higher in patients with ICH, whereas that of NDDs, similar to other neurological complications, did not differ between the two groups (Table 3).

**Table 3:** Comparison between patients with and without ICH NDDs: neurodevelopmental disorders; ^¶^Severity was divided into severe and non-severe, including mild and moderate hemophilia; ^††^Statistical analysis was performed using the chi-square test, and statistical significance was set at p<0.05.

Variable	with ICH (n=5)	without ICH (n=46)	p-value	χ^2^ value
Hemophilia A, n (%)	3 (60.0)	33 (71.7)	0.58	0.30
Severe hemophilia^¶^, n (%)	4 (80)	32 (69.6)	0.63	0.24
Epilepsy, n (%)	2 (40.0)	0 (0)	<0.001^††^	19.15
NDDs, n (%)	1 (20.0)	4 (8.7)	0.42	0.65

The details of the five patients with epilepsy are described below.

Case 1

**Figure 1:**
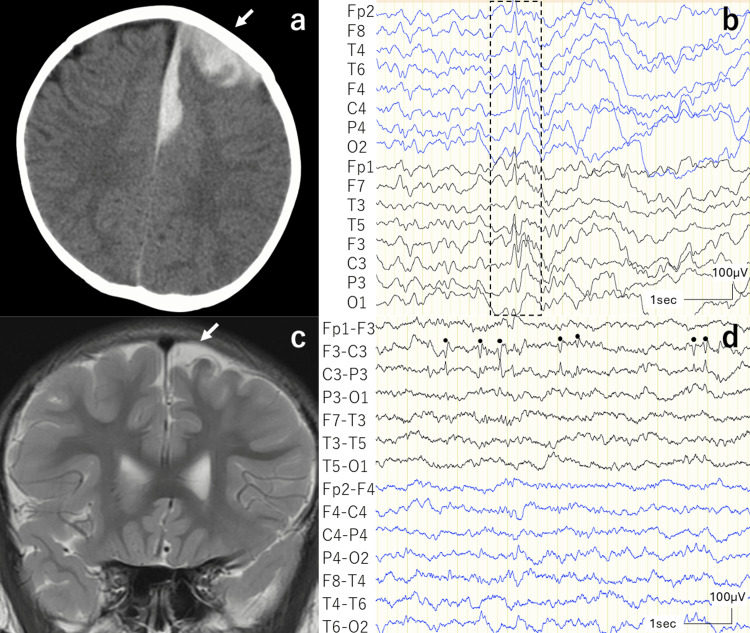
CT, MRI, and EEG findings of Case 1 (a) CT scan at the first convulsion showed subdural hematomas (SDH) in the left frontal lobe (arrow). (b) Referential montage EEG conducted one and a half years after discontinuation of sodium valproate (VPA) showed bilateral frontal spikes (surrounded by the dotted line). (c) Mild atrophy in the left frontal lobe was seen in follow-up MRI (arrow). (d) Bipolar montage EEG at follow-up showed left central spikes (dot).

A nine-month-old male was brought to the emergency room because of febrile status epilepticus. Electroencephalography (EEG) showed no epileptiform discharges; however, computed tomography (CT) revealed an SDH in the left frontal lobe (Figure 1a). Blood examination showed remarkably prolonged activated partial thromboplastin time and a decrease in factor Ⅸ activity, leading to the diagnosis of HB. The patient was treated with factor replacement therapy, and the hematoma progressed without enlargement. During hospitalization, in addition to transient right hemiparesis, the patient had clonic seizures of the right leg even after the fever resolved. He was eventually discharged 17 days after the onset without any symptoms.

The patient was prescribed sodium valproate (VPA) for seizures, which was discontinued a year after discharge because he had no seizures. One and a half years after discontinuation, the patient experienced a seizure induced by fever, and the EEG showed spikes in the bilateral frontal area (Figure 1b); therefore, VPA was resumed. After another three years, follow-up MRI showed mild atrophy in the left frontal lobe (Figure 1c), and EEG abnormalities persisted (Figure 1d). VPA was discontinued when the epileptiform discharges temporarily disappeared. However, EEG abnormalities recurred, afebrile tonic-clonic seizures were observed, and VPA was resumed. Thereafter, the patient continued antiseizure medication and epileptiform discharges finally disappeared at the time after eight years after the onset of epilepsy, and no epileptiform discharges have been observed since then.

Case 2

**Figure 2:**
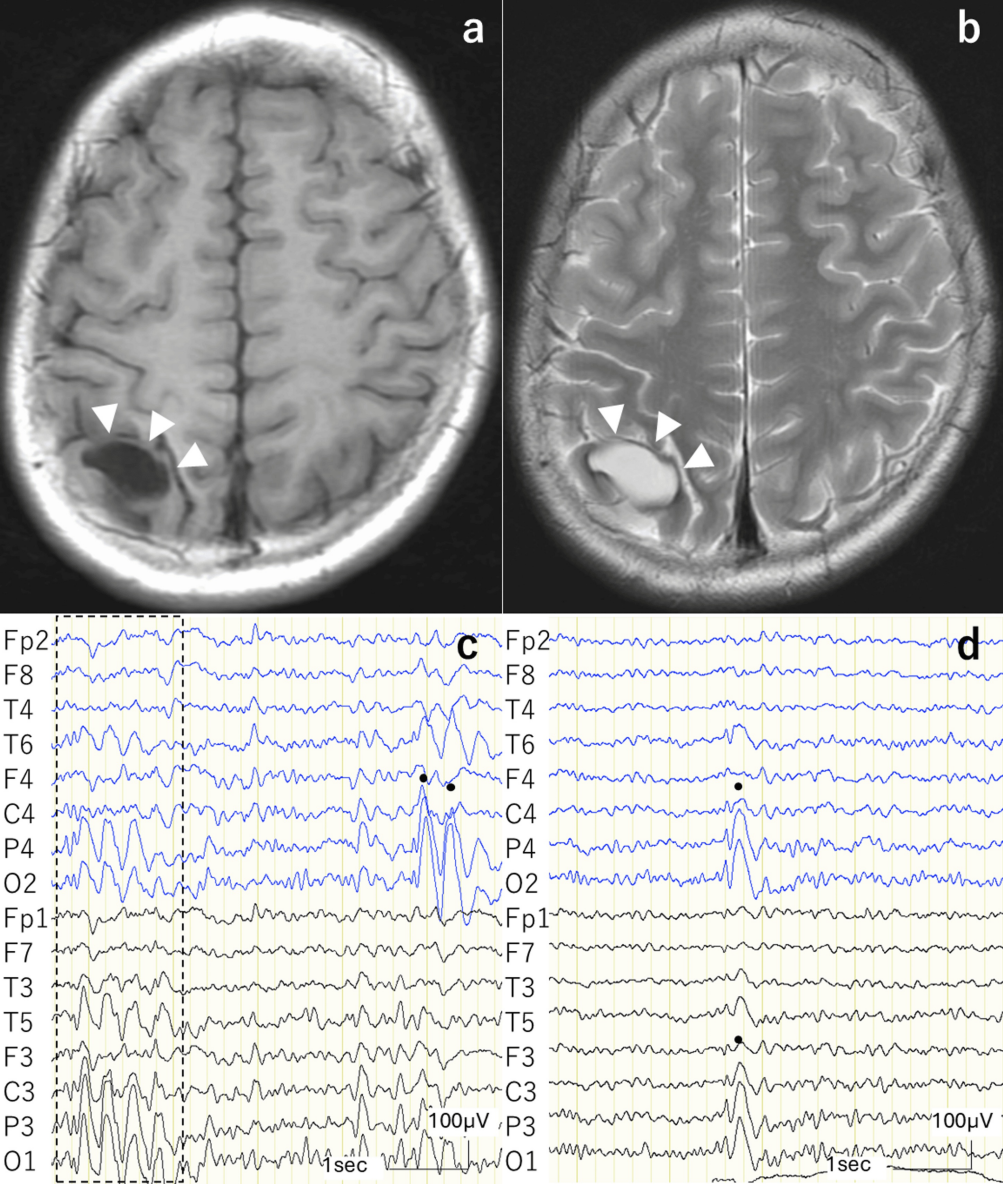
MRI and EEG findings of Case 2 (a) T1 and (b) T2-weighted images show a cystic lesion in the right parietal lobe (arrowhead). The lesion was surrounded by T2 low signal intensity, suggesting hemosiderin deposition. (c) Referential montage EEG in the acute phase showing bilateral parieto-occipital (surrounded by a dotted line) and right parieto-occipital (dot) spike-and-waves. (d) EEG performed three years after the convulsion showed bilateral parieto-occipital spike-and-waves (dots).

A 10-year-old male who had been treated with recombinant factor Ⅸ for HB presented to the emergency room with complaints of afebrile convulsion and cyanosis for a minute. MRI revealed a cystic lesion in the right parietal lobe with low signal intensity on T1-weighted images and high signal intensity on T2-weighted images (T2WI), surrounded by low signal intensity on T2WI (Figures 2a, 2b). This signal pattern indicated that the lesion was a cyst with surrounding hemosiderin deposition, suggesting an old hemorrhage. EEG showed epileptiform discharges, mainly in the bilateral parieto-occipital and right parietal areas (Figure 2c). No recurrence of seizures or apparent epileptic discharges was observed on the EEG after VPA treatment. The patient was followed up without changing the medication dose, and blood concentration decreased as weight increased. EEG abnormalities were observed three years later (Figure 2d). After the VPA dose was adjusted, no definite epileptiform discharge was observed four years after epilepsy onset.

Case 3

**Figure 3:**
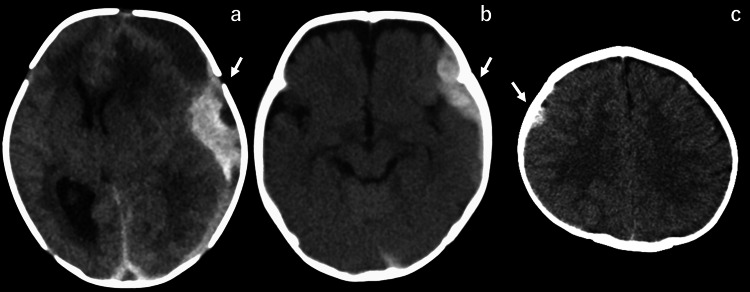
CT findings of Cases 3, 4 and 5 CT images from (a) Case 3, (b) Case 4, and (c) Case 5 show SDH in the left parietal lobe, left temporal lobe, and right frontal lobe (arrows), respectively.

A full-term male neonate, delivered by cesarean section, presented with poor feeding, lethargy, and a bulging anterior fontanelle on the second day of life. CT revealed an SDH in the left parietal lobe, with a midline shift (Figure 3a). Hematoma evacuation was performed via craniotomy. Blood tests showed a decrease in factor VIII activity below 1%. The patient was diagnosed with HA and treated with factor replacement therapy. During hospitalization, the patient experienced no seizures and was discharged 70 days after the onset of hemorrhage without any neurological symptoms.

Case 4

A four-month-old male infant presented to the emergency room with two days of fever, lethargy, recurrent vomiting, and a bulging anterior fontanelle. A CT scan revealed an SDH in the left temporal lobe (Figure 3b). Blood tests showed a decrease in factor VIII activity to 2.8%. The patient was diagnosed with HA and treated with factor replacement therapy without the need for surgery. No enlargement of hematoma was seen. After confirming the resolution of the hematoma, the patient was discharged 12 days after the onset of symptoms without any neurological issues.

Case 5

A 13-month-old male infant presented with febrile status epilepticus. A CT scan revealed an SDH in the right frontal lobe (Figure 3c). Blood tests showed a decrease in factor VIII activity below 1%. The patient was diagnosed with HA and treated with factor replacement therapy, with no evidence of hematoma enlargement. During hospitalization, the patient experienced a seizure three days after the onset but had no recurrence. He was discharged 31 days after the onset of symptoms without any neurological issues.

## Discussion

In this study, no statistically significant neurological complications, except for epilepsy, were observed in the five patients with ICH. This indicates that our cohort might have a favorable prognosis compared to previous reports in which 16.6% to 58.6% of patients with hemophilia and ICH had developmental delay and 12.5% to 44.8% had paralysis [10,11]. In contrast, two of the five patients had epilepsy, suggesting that epilepsy can develop even in patients without other severe neurological sequelae. The association between epilepsy and ICH has been well documented in studies of post-traumatic epilepsy (PTE). In these studies, SDH was consistently noted to be a risk factor for PTE, while other sites, such as the intraventricular or subarachnoid, were inconsistent [7-9]. In our study, Case 1 had SDH without parenchymal injury, and Case 2 was considered to have had an intraparenchymal hemorrhage. SDH contributes to the development of epilepsy through the toxic effects of hemoglobin breakdown products [12]. Therefore, SDH itself can be a risk factor even without parenchymal injury, like Case 1. The proportion of SDH among ICH in patients with hemophilia has been reported to be as high as 26.8% to 44.2% [5,13,14], and even mild head trauma can easily cause SDH in patients with hemophilia [15]. In our study, all four patients with a clinical history of ICH were diagnosed with SDH. Considering the above, careful follow-up of epilepsy is required in the management of patients with hemophilia for intraparenchymal hemorrhage and SDH alone. The etiology of epilepsy is useful information in selecting treatment options, including second-line therapies.

In this study, 9.8% of patients had ICH, which is relatively high compared to previous reports on pediatric hemophilia, ranging from 2.1% to 10.8% [10,11,13,16]. Severe hemophilia, age (<three years or>40 years), and insufficient prophylaxis are known risk factors for ICH [5,10,11,13,17]. Our patients with ICH were considered to be in the high-risk group because four patients had severe hemophilia, and all cases in which the age of onset was identified were under two years. In addition, all our patients with a clinical history of ICH had an onset before the diagnosis of hemophilia, suggesting that the lack of prophylaxis at the time of hemorrhage could be a reason for the high frequency of ICH. The association between the hemophilia type and hemorrhage remains controversial. Studies have suggested that HA is more severe than HB due to a higher risk of nontraumatic soft tissue hematoma, joint arthroplasty, and higher factor consumption [18-20]. However, several reports have indicated that the risk of ICH did not differ between HA and HB [5,10,13,17], and these results are consistent with those of our study. Although the presence of an infusion factor inhibitor is considered a risk factor for hemorrhage [5,13,17], none of the patients in our study had an inhibitor at the time of bleeding.

This study had several limitations. Although the prevalence of ICH in this study was within the previously reported range, there remains a possibility of inaccuracy owing to the small sample size. In addition, a selection bias may exist because this was a single-center study. Imaging studies were not performed on all patients; as such, asymptomatic ICH cases may have been misclassified in the without ICH group. A detailed assessment of the characteristics of epilepsy in patients with hemophilia is difficult because of the small sample size.

## Conclusions

Epilepsy resulting from ICH can develop in pediatric patients with hemophilia even in the absence of severe neurological abnormalities such as developmental delay or paralysis. In addition, patients with hemophilia are more likely to have SDH, a well-known risk factor for epilepsy. Understanding the etiology of epilepsy is crucial, as it influences treatment options, efficacy, and prognosis. Our findings indicate that imaging studies are warranted in patients with hemophilia and epilepsy, even when other neurological symptoms are not evident, as epilepsy can occur as the sole neurological symptom in cases complicated by ICH.
